# Evaluation of the immunochromatographic assay for HTLV-1/2 detection in Gabon: genotype-specific limitations in Central Africa

**DOI:** 10.1128/spectrum.03867-25

**Published:** 2026-03-23

**Authors:** Eldridge Fedricksen Oloumbou, Hanneke Fanhole Moussonda-Mouele, Abdoulaye Diané, Michel Charonne Mbezele-Ba-Nsegue-Abessolo, Ivan Mfouo-Tynga, Augustin Mouinga-Ondémé

**Affiliations:** 1 Unité des Infections Rétrovirales et Pathologies Associées, Centre Interdisciplinaire de Recherches Médicales de Franceville (CIRMF) 89249, Franceville, Gabon; University of Texas Medical Branch at Galveston, Galveston, Texas, USA

**Keywords:** HTLV-1/2, Rapid Diagnosis test, immunochromatographic assay, central African genotypes, sensitivity

## Abstract

The human T lymphotropic virus types 1 and 2 (HTLV-1/2) are highly endemic in sub-Saharan Africa but are largely unreported. This is due to limited knowledge of HTLV-1/2 infections and a lack of suitable facilities for systematic screening. To address this, the flow immunochromatographic assay (IC) was developed for the rapid, point-of-care detection of anti-HTLV-I/II antibodies in blood samples, offering a convenient tool for improving healthcare in low-income countries. Previous reports on the IC assay have noted high specificity (up to 99.42%) and sensitivity (100%) for certain HTLV-1 strains. In this study, we assessed the performance of the IC assay using HTLV-1/2 samples from Gabon and compared its results with those obtained using two reference serological methods: enzyme-linked immunosorbent assay (ELISA) and western blot (WB). Our findings indicate that the overall performance of the IC assay was lower with Gabon-related strains, showing a sensitivity of 66.7% (95% CI, 58.5%–74.3%) and a specificity of 99.1% (95% CI, 96.7%–99.8%). A notable variation in sensitivity was observed across different HTLV-1 genotypes: B-genotype with 73.7% sensitivity (95% CI, 61.0%–83.3%), D-genotype with 25% sensitivity (95% CI, 0.04%–0.7%), and A-genotype with 100% sensitivity (95% CI, 0.7%–100%). The IC assay also showed a positive reaction with samples of the rare F-genotype and correctly detected all eight samples related to HTLV-1-associated diseases that were tested. These results suggest that while the IC assay appears suitable for detecting A-genotypes, it has relatively lower sensitivity for the B- and D-genotypes that are common in Central Africa. Therefore, IC test outcomes, especially negative results, should be interpreted with caution in this region.

**IMPORTANCE:**

Over 5 million people in Africa are living with HTLV-1 without knowing their serological status or the potential risks of associated diseases. Contrary to other industrialized countries, where systematic HTLV-1/2 screening is implemented, HTLV-1 antibody screening appears to be totally absent in low-income countries of the African continent. Such conditions are undoubtedly due to the high cost of current HTLV-1/2 reagents and laboratory equipment needed for retrovirus screening. In this context, the newly developed lateral flow immunochromatographic assay (IC), specifically the MP Diagnostics ASSURE HTLV-I/II Rapid Test, could help not only to gain a better understanding of HTLV-1/2 epidemiology but also to improve the health care system of low-income countries. Thus, in this study, we aimed to assess the performance of this IC assay with HTLV-1/2 African genotypes.

## INTRODUCTION

The human T-cell lymphotropic virus type 1 (HTLV-1) was the first oncogenic agent to be isolated from humans and causes severe hematologic cancers (1). Globally, an estimated 5 to 10 million people are infected, primarily within endemic regions, such as Japan, the Caribbean basin, South America, Iran (Mashhad region), the Australo-Melanesian region, and sub-Saharan Africa (SSA) (2, 3). For a small percentage of HTLV-1-infected people, serious medical conditions can develop after a long latency period. Adult T-cell leukemia/lymphoma (ATL): about 3% to 7% of infected individuals may suffer from this fatal T-cell lymphoproliferation. The HTLV-1-associated myelopathy/tropical spastic paraparesis (HAM/TSP) is a smaller group, less than 1% to 3% of infected individuals, who may experience a disabling condition with progressive spastic weakness of lower limbs and no remission (4–7). A recent review conducted by the World Health Organization (WHO) has further highlighted the gravity of HTLV-1 infection. The review found that people living with HTLV-1 have increased mortality and that the virus is linked to at least 16 different diseases (8). The primary routes of HTLV-1 transmission are vertical (mother-to-child via prolonged breastfeeding), sexual (predominantly male-to-female), and parental (contact with infected blood products) (9–13). A significant association between TSP/HAM and blood transfusion has been noted, with a higher percentage of TSP/HAM patients (20%) reporting a history of blood transfusion compared to control groups (3%–5%) (7, 14).

Similarly, ATL appears to occur predominantly among individuals with a history of childhood HTLV-1 transmission (15). These findings underscore the importance of implementing systematic HTLV-1 screening for blood donors and pregnant women, especially in regions endemic to this oncogenic virus. Systematic HTLV-1 screening of blood donors and pregnant women has been established in Japan, certain Caribbean territories, Seychelles, and more recently in Brazil, where this oncogenic virus is endemic.

Surprisingly, in most SSA countries considered highly endemic for this retrovirus, this screening strategy is not mandatory. This observation can be attributed to two primary reasons. First, the majority of the medical staff possesses insufficient knowledge of the virus and its related diseases. Second, the cost of most currently available HTLV-1/2 diagnostic and confirmatory tests, including enzyme-linked immunosorbent assay (ELISA), western blot (WB), and polymerase chain reaction (PCR), is prohibitive (16), rendering them impractical for routine screening. In response to these challenges, Teoh et al. have recently developed the “MP Diagnostics ASSURE HTLV-I/II Rapid Test,” a rapid immunochromatographic assay (IC) for the detection of anti-HTLV-I/II antibodies in serum, plasma, and whole blood specimens (17).

The development of innovative diagnostic tools offers a promising path to improving healthcare in resource-limited settings, such as those found in SSA. The IC assay, a point-of-care test for HTLV-1/2, has previously reported high sensitivity (99.4%) and specificity (100%) with HTLV-1 strains from MP Biomedical laboratory and Pitié Salpêtrière Hospital (Paris, France). However, in the context of the African sub-Saharan region, known as highly endemic for several infectious circulating diseases, as well as the existence of the greatest HTLV-1 genetic diversity, the research question was to know if this IC assay, named Assure HTLV-I/II Rapid Test, could reliably detect HTLV-1/2 infections, specifically with samples from Gabon. Therefore, this study was designed to evaluate the accuracy of the MP Diagnostics ASSURE HTLV-I/II Rapid Test against HTLV-1 genotypes mainly found in Gabon, specifically, and in the SSA region in general, by comparing its diagnostic performance with established reference serological methods (ELISA and WB).

## RESULTS

### Overall performance of the ASSURE HTLV-I/II Rapid Test on Gabonese samples

Analysis of the stand-alone readings from raters indicated a complete absence of discordant results with the IC assay. Samples were consistently categorized as positive (exhibiting varying signal intensities: +, ++, or +++) or negative by both independent raters. This high degree of consistency resulted in a strong agreement between raters (Kappa value = 97.8%) regarding the interpretation of the ASSURE HTLV-I/II Rapid Test outcomes.

In samples from Gabon with WB confirmed HTLV-1 and HTLV-2 infections, the Assure HTLV-I/II Rapid Test demonstrated an overall specificity of 99.1% (95% CI, 96.7%–99.8%). Its sensitivity (excluding WB indeterminate samples) was 66.7% (95% CI, 58.5%–74.3%). For samples with HTLV-1, HTLV-2, and false positive WB profiles, the positive predictive value (PPV) was 65.7% (90/137). The negative predictive value (NPV) of Assure HTLV-1/2 Rapid Test, calculated for samples with negative ELISA and IC results, as well as WB-positive samples that were IC-negative, was 83.4% (216/259) (Table 1).

**TABLE 1:** Total diagnostic performance of ASSURE HTLV-I/II Rapid Test

Diagnostic performance	Performance	95% CI^*a*^
Sensitivity	66.7% (90/135)	58.5%–74.3%
Specificity	99.1% (216/218)	96.7%–99.8%
Positive predictive value	65.7%	57.4%–73.1%
Negative predictive value	83.4%	78.4%–87.4%
Kappa value	0.978	0.945–0.982

^*a*^ CI, confidence interval.

### Evaluation of the specificity of the IC assay

To evaluate the specificity of the IC assay, a total of 218 plasma samples were tested. These samples were categorized into three groups: 218 plasma samples that were confirmed as seronegative for HTLV-1/2 infection by ELISA. Samples that were reactive by ELISA but negative by WB. Samples with indeterminate results by WB and not amplified by PCRs (Env and Tax/Rex). Group 1 (seronegative by ELISA): out of the 218 seronegative samples, two samples (from adult general population) showed a positive reactivity with the IC assay. These two samples displayed an intensive reactivity (+ and +++). Based on these results, the specificity of the IC assay for this group was estimated to be 99.1% (216/218, 96.7%–99.8%) (Table 2). For groups 2 and 3 (reactive by ELISA with negative/indeterminate WB/PCR), no reactivity was observed with the IC assay for the samples in these two groups. Therefore, the specificity of the IC assay for these samples was estimated to be 100% (88/88, 95.8%–100%).

**TABLE 2:** Evaluation of the specificities of the IC assay^*b*^

	Sample origin	N	HTLV-1/2 Rapid Test	
			NEG (%)	95% CI
ELISA (−)	Blood donors	60	60/60 (100)	93.9–100.0
	Pregnant women	38	38/38 (100)	90.8–100.0
	Patients	20	20/20 (100)	83.9–100.0
	Rural adult population	100	98/100 (98)	93.0–99.5
	Total	218	216/218 (99.1)	96.7–99.8
WB (NEG) + WB (IND)^*a*^	Blood donors	29	29/29 (100)	88.3–100
	Pregnant women	14	14/14 (100)	78.4–100
	Patients	5	5/5 (100)	56.5–100
	Rural adult population	40	40/40 (100)	91.2–100
	Total	88	88/88 (100)	95.8–100

^*a*^ WB (IND): including only samples whose WB profile was indeterminate with a negative result by PCR.

^*b*^ N, total number of samples included in each type of item; WB (NEG), plasma sample displayed both reactivity by ELISA and a negative WB profile.

### Sensitivity of IC assay according to the western blot profile

The overall sensitivity of IC assay for the detection of anti-HTLV-1 antibodies was 67.9% (89/131). This sensitivity varied from 52% to 81% depending on the population assessed (pregnant women, blood donors, inpatients, adults from rural communities) (Table 3). The highest sensitivity was observed in samples from inpatients (17/21, 80.9%). For HTLV-1, only one out of the four samples with an HTLV-2 WB profile reacted positively (++) with the IC assay, resulting in a sensitivity of 25.0% (1/4). Among eight samples with an HTLV WB profile, only one tested positive with the HTLV-1/2 IC assay. This resulted in an estimated sensitivity of 12.5% (1/8) for samples with this profile. Of note, two of these eight samples had been previously tested and amplified for the 522-bp fragment of HTLV-1 Env gene. Finally, among 49 plasma samples with an indeterminate WB profile, only two were considered positive for HTLV infection based on the amplification of the 210-bp fragment from Tax/Rex gene by PCRs. Unfortunately, neither of these two positive samples displayed a positive reactivity with the HTLV-1/2 Rapid Test.

**TABLE 3:** Sensitivity of the IC assay according to WB profiles^*a*^

		Samples origin				
	WB profiles	Blood donors	Pregnant women	Inpatients	Rural adult population	Total
	HTLV-1 (%)	29 (22.1)	20 (15.3)	21 (16.0)	61 (46.6)	131 (100)
HTLV-1/2 Rapid Test	NEG	14	5	4	19	42
	+	4	11	10	20	45
	++	5	3	5	11	24
	+++	6	1	2	11	20
	*n*+/N (%)	15/29 (51.7)	15/20 (75.0)	17/21 (80.9)	42/61 (68.9)	89/131 (67.9)
	95% CI	33.5%–69.9%	56.0%–94.0%	64.1%–97.7%	57.0%–80.4%	59.9%–75.9%
	HTLV-2 (%)	1 (25.0)	0 (0)	0 (0)	3 (75.0)	4 (100)
HTLV-1/2 Rapid Test	NEG	1	–^*d*^	–	2	3
	++	0	–	–	1	1
	*n*+/N (%)	0/1 (0)	–	–	1/3 (33.3)	1/4 (25.0)
	95% CI	0.0%–79.3%	–	–	6.1%–79.2%	0.04%–0.7%
	HTLV (%)	0 (0)	4 (50.0)	2 (25.0)	2 (25.0)	8 (100)
HTLV-1/2 Rapid Test	NEG	–	3	2	2	7
	+	–	1	0	0	1
	*n*+/N (%)	–	1/3 (33.3)	0/2 (0)	0/2 (0)	1/8 (12.5)
	95% CI	–	4.6%–69.9%	0.0%–65.8%	0.0%–65.8%	2.2%–47.1%
	IND (%)					
HTLV-1/2 Rapid Test *n*+/N (%)	NEG^*b*^	17	8	0	22	47
	POS^*c*^	0	0	2	0	2
	*n*+/N (%)	0/17 (0)	0/8 (0)	0/2 (0)	0/22 (0)	0/49 (0)
	95% CI	0.0%–18.4%	0.0%–32.4%	0.0%–65.7%	0.0%–14.9%	0.0%–7.3%

^*a*^ *n*+, number of samples displaying positive reactivity by using HTLV-1/2 Rapid Test; N, total number of included samples per item; NEG, samples which did not display positive reactivity with HTLV-1/2 Rapid Test; IND, indeterminate WB profiles.

^*b*^ NEG: samples that displayed an indeterminate WB profile with negative PCRs.

^*c*^ POS, samples that displayed an indeterminate WB profile with positive PCRs; CI, confidence interval.

^*d*^ –, no data are available.

### Sensitivity of the HTLV-1/2 Rapid Test on HTLV-1 genotypes

To evaluate the reactivity of the HTLV-1/2 Rapid Test (RDT) with different viral genotypes, we analyzed plasma samples from 72 of the 131 HTLV-1-positive individuals included in this work, whose HTLV-1 subtypes had been previously characterized at the molecular level (Table 4). The results showed varying reactivity rates among the different genotypes. The highest reactivity was observed with plasma samples from individuals infected with HTLV-1 subtype A, with a 100% reactivity rate (9/9). For subtype B, which is common in Central Africa, the reactivity rate was estimated at 73.7% (42/57). The lowest reactivity was observed with plasma samples from individuals infected with HTLV-1 genotype D, at 25% (1/4). Additionally, the two plasma samples from individuals infected with HTLV-1 genotype F reacted with a low intensity (+).

**TABLE 4:** Evaluation of the reactivity of HTLV-1 subtypes^*a*^

	HTLV-1/2 Rapid Test					95% CI
Genotypes	NEG	+	++	+++	*n*+/N (%)	
HTLV-1B	15	21	9	12	42/57 (73.7)	61.0%–83.3%
HTLV-1A	0	3	6	0	9/9 (100)	0.7%–100.0%
HTLV-1D	3	0	0	1	1/4 (25)	0.04%–0.7%
HTLV-1F	0	2	0	0	2/2 (100)	0.3%–100.0%
Total	18	26	15	13	54/72 (75)	63.9%–83.5%

^*a*^ *n*+, number of samples displaying positive reactivity by using HTLV-1/2 Rapid Test; N, total number of samples included per item; NEG, samples retained as negative by using HTLV-1/2 Rapid Test; CI, confidence interval.

### Sensitivity of the IC-assay with samples from inpatients with HTLV-1-associated diseases

The HTLV-1 Rapid Test showed a 100% reactivity rate in samples from patients with HTLV-1-related diseases. However, the intensity of the reaction was low in 37.5% (3/8) and medium in 50% (4/8) of the cases (Table 5). Furthermore, we noticed that all four tested samples from inpatients suffering from HTLV-1-associated diseases (ATL, TSP/HAM, and/or HTLV-1-associated inflammatory myopathy [HAIM]) and infected with HTLV-1 genotype B were successfully detected by the IC assay.

**TABLE 5:** Evaluation of the IC assay with samples from inpatients with HTLV-1 related diseases^*a*^

		HTLV-1/2 Rapid Test				Identified genotypes	
Samples origin	Number	+	++	+++	*n*+/N	A	B
ATL	2	1	1	0	2/2	–^*b*^	2
TSP/HAM ± HAIM	5	2	3	0	5/5	4	1
HAIM	1	0	0	1	1/1	–	1
Total	8	3	4	1	8/8	4	4

^*a*^ *n*+, number of samples displaying positive reactivity by using HTLV-1/2 Rapid Test; N, total number of samples included per item; HAIM, HTLV-1-associated inflammatory myopathy.

^*b*^ –, no data are available.

## DISCUSSION

The African continent is considered the largest HTLV-1 endemic region, with at least 5 million people living with HTLV-1 (2). While this number seems surprising, it is considered an underestimation due to the lack of available HTLV-1 epidemiological data for most African countries (3). Indeed, testing for HTLV-1 is scarce in the African region, and the need for novel epidemiological data has been pointed out as a priority by the WHO (18). Point-of-care tests for HTLV-1/2 antibodies, which do not require laboratory equipment, would be a welcome development to facilitate testing uptake. This would both improve the understanding of HTLV-1 epidemiology and allow for the identification of those living with the virus to mitigate its transmission. In Gabon, Central Africa, HTLV-1 prevalence ranges from 7% to 9% among adult rural populations. Central Africa is also a region with the highest diversity of HTLV-1 circulating genotypes. In contrast to other regions where HTLV-1A is the most common HTLV-1 subtype, the main genotype in Central Africa is HTLV-1B (12, 19). However, other genotypes, including A, D, E, F, and G, are also present but rare in the region (3, 20). Given this context of viral diversity and the high endemicity of other pathogens that may cause cross-reactivity in antibody-based assays, it is of utmost importance to validate diagnostic tests in the region.

The aim of this study was to evaluate the performance of MP Diagnostics ASSURE HTLV-I/II Rapid Test using plasma samples from Gabon. If the purpose of this study appears at first glance limited to an African-specific geographic region, in the current context of globalization, the research question developed in this study could also be relevant for other parts of the world where this IC assay could be used. Indeed, remember that HTLV-1 infected more than 5 to 10 million people worldwide, with a heterogeneous distribution: Asia, America, Europe, Australo-Melanesia, and Africa (2, 3, 21). In Europe, for example, where HTLV-1/2 infection is known as non-endemic, most people reported to be infected seem to have an African origin or reported having experienced an African partner (2, 22). In South America, particularly in Brazil, the circulation of the main central African genotype: HTLV-1B, and precisely the SF26 prototype strain (23) was reported. Moreover, a recent systematic review and meta-analysis indicated that HTLV-1 is present among immigrants and refugees in various countries of the world, with an observed combined prevalence of approximately 1.28% (24). Which reinforces the idea that the circulation of HTLV-1 strains is no longer limited to specific regions of the world. Thus, considering these data, the results of this work produced in an African context could undoubtedly present a great general interest in the case of using this IC assay.

Herein, we observed an excellent specificity of 99.1% (216/218, Table 2) but low sensitivities for HTLV-1 (67.9%, 89/131, Table 3) and HTLV-2 (25%, 1/4, Table 3). While the specificity was comparable to a previous validation study by Teoh et al., our observed sensitivity was 1.5-fold lower than their reported 99.42%. That study used samples from MP Biomedicals Asia Pacific (Singapore) and a laboratory in Paris, France. This difference in sensitivity could be attributed to the different HTLV-1 genotypes present in the study populations. The ASSURE HTLV-I/II Rapid Test principle is primarily based on the detection of total antibodies to HTLV-I/II using a colloidal gold-labeled recombinant trifusion antigen (MGK). This antigen was developed using strains from Singapore, where the cosmopolitan genotype A is most common (17, 25, 26). In contrast, our samples were from a region with different circulating strains. Previous data from the analysis of the 522-bp fragments encoding the gp46/GD21 genes showed that West and South African strains are closely related (97% to 99%) to the cosmopolitan HTLV-1A subtype (27). However, Central African HTLV-1B strains are more closely related (95.6%–99.4%) to the genotypes B strains (EL and Z15) from Zaïre (now the actual Democratic Republic of Congo).

Moreover, a study by Capdepont et al. analyzed the entire env gene (1,467-bp long fragment) of 65 HTLV-1 isolates from Gabon, French Guiana, West Indies, and Iran. The analysis highlighted the existence of a genetic drift ranging from 0.68% to −2.66% (28). Interestingly, the study reported that about 70% (108/164) of the observed mutations were localized in the fragment coding for the gp46 glycoprotein, with 30% of the whole observed mutations leading to a change in the amino acid sequence. Specifically, for the 33 isolates from Gabon, these researchers noticed a nucleotide divergence of nearly 3% between strains, with 114 mutation sites widely spread along the env gene. Furthermore, 35% (26/74) and 25% (10/40) of these observed mutations led to some amino acid substitutions for fragment encoding the gp46 and GD21 glycoproteins, respectively. It is well known that a protein’s amino acid sequence directly influences its three-dimensional structure, which plays a central role in viral penetration and the host immune response. As a result, based on these previous data, such genetic drift could explain the observed differences in assay sensitivity when testing samples from people infected by different HTLV-1 strains.

However, although these cited papers confirm Env mutations in African strains, they do not demonstrate HTLV-1B-specific and high-frequency amino acid mutations in the antigenic regions of the two HTLV-1 antigens used in the IC assay (MTA-1 and GD21). The main point is that simply citing papers that confirm Env mutations in African strains is insufficient to explain the assay’s reduced sensitivity (73.7%), because those papers do not demonstrate HTLV-1B-specific and high-frequency amino acid mutations within the specific antigenic regions used in your IC assay: MTA-1 (Env 162-209) and GD21 (Env 361-404). The reduced sensitivity is likely due to unknown and frequent mutations within the Env antigenic recognition sites because the genetic analysis of HTLV-1 strains circulating in Gabon has not yet reached sufficient numbers. In summary, the new hypothesis shifts the focus from known, published, but infrequent mutations to the possibility of numerous unknown amino acid mutations in the IC assay’s targets, stemming from under-sampling in regional genetic studies, such may exist and contribute to the reduced sensitivity noted.

Our results reinforce this hypothesis that the IC assay demonstrated 100% sensitivity for infection with the cosmopolitan HTLV-1A subtype; the sensitivity with HTLV-1B genotypes was considerably lower (73.7%). Furthermore, the IC assay successfully detected both individuals infected with HTLV-1F subtype, yet only 1/4 of the samples infected with HTLV-1D genotype were positive. These findings highlight the necessity of further studying and better understanding the nucleotide divergence among all HTLV-1 genotypes. Such genetic differences must be considered when developing future assays for HTLV-1.

Another possible explanation for the observed results could be the shelf life of samples and the number of freeze-thaw cycles. However, a logistic regression analysis showed no association between the outcomes of the IC assay and the shelf life of samples included in this study (*P* value = 0.6378). Furthermore, none of the samples used in this study were subjected to multiple freeze-thaw cycles. Therefore, it is unlikely that sample viability was a factor in the low sensitivity observed.

The result of 100% of the positive samples with the IC assay from individuals with HTLV-1-related diseases (Table 5), regardless of the HTLV-1 genotype, could be due to high levels of both HTLV-1 proviral load and anti-HTLV-1 antibodies in HTLV-1 samples. This finding not only facilitates the diagnosis of HTLV-1 in symptomatic patients but also highlights the need to include people with a wide range of clinical characteristics and disease progression when validating diagnostic tests for HTLV-1.

The HTLV-2 infection is less prevalent than HTLV-1, and a low number of cases of HTLV-2-associated diseases have been reported globally. In Gabon, HTLV-2 prevalence is about 6- to 73-fold lower than HTLV-1 prevalence, and in this disparity lies the scarcity of usable HTLV-2 (12, 19). Most HTLV-2-infected people are co-infected with HTLV-1, making any HTLV-2 mono-infected individual (positive sample) inherently difficult to find. Thus, using plasma isolated from HTLV-1/HTLV-2 co-infected individuals to assess the performance of the assay about the HTLV-2 infection could clearly induce a bias in the analysis. Only four samples isolated from HTLV-2-infected people were available to assess the performance of the IC assay. This prevents any reliable conclusion from being formulated. Among these four HTLV-2-infected samples, only one was positive with the IC assay, suggesting a hypothetically low sensitivity for HTLV-2. As for HTLV-1, this result could probably relate to the existence of genetic diversity. Similar to HTLV-1, this result could likely be attributed to genetic diversity. The HTLV-2 has four distinct genotypes (A, B, C, and D), all of which appear to be related to the geographic origin of infected individuals. While genotypes A and B are found in the Americas and Europe, genotypes C and D are considered to be from Brazil and Central Africa, respectively (29). Thus, future studies that consider the genetic drift of HTLV-2 are necessary to better assess the sensitivity of this IC assay.

Besides these probable explanations, environmental conditions like temperature, humidity, and storage duration of the IC assay should also be considered. In other words, although these devices could be easily stored at room temperature, temperature and humidity fluctuations experienced by the IC tests during their transport from Asia to Gabon could also influence the performance of the test. Unfortunately, in this work, these parameters were not evaluated. Thus, it would be interesting to consider these factors in other future investigations and similar studies, which could be held elsewhere, particularly in other countries of the sub-Saharan region of Africa.

It is important to remember that using such medical laboratories’ tools, which allow passing around 40% of HTLV-1 positive, could pose a real danger to public health, mainly in the case of transfusion safety. Indeed, the risk of HTLV-1 transmission via blood transfusion is one of the most critical public health challenges (30, 31). Previously, it has been demonstrated that HTLV-1 acquired through transfusion increases the risk of a more rapid progression to TSP/HAM compared to other transmission routes (13, 14, 32). For example, it was previously highlighted in Japan that up to 39% (9/23) of patients suffering from TSP/HAM reported a story of blood transfusion 6 months to 2 years before the onset of symptoms, compared to the control groups (14). Furthermore, note that until nowadays, TSP/HAM therapy remains disappointing and discouraging (7, 33). Based on all these data, this IC assay should be absolutely used at the same time with another further sensible and/or specific diagnostic tool to confirm the sample’s outcome, mainly when the analyzed sample is from an individual of African origin. Another solution could be the development of antigenically adapted versions of the Assure HTLV-I/II Rapid Test, including Sub-Saharan African HTLV-1/2 variants. This option could undoubtedly offer better results with strains of this part of the African continent, which will increase the reliability of the test, regardless of where in the world it is used.

We reported an overall specificity estimated at 99.1% (Table 2), like that 100% specificity previously reported by Teoh et al. for the same HTLV-1/2 Rapid Test. Similarly, the excellent analytical specificity reported in this work (100%; Table 2) reinforces the excellent specificity reported for this assay, which has now been confirmed in samples from Central Africa. Indeed, in their previous study, many potential cross-reactive samples and interfering substances were tested with this IC assay, and none showed cross-reactivity with the trifusion antigen (17).

In conclusion, MP Diagnostics ASSURE HTLV-I/II Rapid Test showed excellent overall specificity and performed very well with samples infected with the HTLV-1A genotype. However, the IC assay remained unreliable when it was tested with samples from people infected with strains circulating in Central Africa, especially HTLV-1B and HTLV-1D. Therefore, given its reduced sensitivity toward these genotypes, the ASSURE HTLV-I/II Rapid Test should not be used as a stand-alone screening tool for blood transfusion, particularly in Central Africa; confirmatory or complementary assays are required. However, for manufacturers, it would also be important to consider HTLV-1 antigens from a range of different variants when developing diagnostic assays. Rapid point-of-care tests like ASSURE HTLV-I/II Rapid Test have the potential to be game-changers for routine HTLV-1 routine testing in low-income countries, where this retrovirus remains both highly endemic and a neglected infection. Nevertheless, our findings underscore the critical importance of validating these diagnostic tests within the target regions, even if some of our genotype-specific sensitivity data should be interpreted with caution due to the limited number of cases per group.

## MATERIALS AND METHODS

### Study design and population

This study followed STARD guidelines.

It is a cross-sectional, retrospective study using randomly selected storage samples of plasma from four different populations from Gabon: inpatients with clinical suspicion of HTLV-1 related diseases; pregnant women; blood donors; and adult general rural population. Written informed consent was obtained from all participants and/or their authorized representative. All these samples were previously obtained during studies led in Gabon (12, 34) on HTLV-1/2 infection and screened for HTLV-1/2 antibodies by ELISA (HTLV-I/II ELISA 4.0, MP diagnostic), followed by confirmation using western blot (HTLV BLOT 2.4, MP diagnostic) in samples with a reactive result at ELISA. Similarly, PCRs were previously used both to confirm the positivity to HTLV-1/2 infection for samples with a WB indeterminate profile and to characterize the HTLV-1 genotype. Overall, 451 plasma samples were included in this evaluation (Table 6). Each sample included was collected, separated, and conserved at −80°C as previously described (12, 34), in the biobank of the Interdisciplinary Medical Research Center of Franceville (CIRMF). The average shelf life of samples was 6.2 years, and for all included samples, the maximum number of freeze-thaw cycles was only one. Table 6 summarizes the complete process from the moment blood draw to long-term storage at the biobank facility at CIRMF.

**TABLE 6:** Summary of the full process from the moment blood draw to long-term storage at the biobank facility at CIRMF

Step	Detail	Purpose/context
Collection	5–7.5 mL of blood in EDTA tubes	EDTA is an anticoagulant, preserving the sample integrity for plasma separation
Initial processing	Centrifuged at 2,000 rpm for 15 min within 12 to 24 h of sampling	Separates plasma and cellular components (buffy coat). The time window minimizes sample degradation
Separation	1.5–2 mL of plasma and 1–1.5 mL of buffy coat separated using single-use Pasteur pipettes	Ensures sterility and consistency in volume separation
Initial freezing (temporary)	Firstly frozen at −20°C at CHUL for around 2 weeks	This is the initial freeze-thaw cycle mentioned in the inclusion criteria
Long-term storage	Transferred to CIRMF facilities and stored at −80°C until analysis	Standard ultra-low temperature for long-term biobanking and preservation of biological materials

Globally, the undertaken sampling approach was to evaluate the assay’s performance against different HTLV serological types confirmed by WB:

Sampling targets by WB profile:

- HTLV-1 WB profiles: a detection rate of 20%–40% of samples.
- Seronegative by ELISA: targeted at around 50%–60% of samples.
- HTLV-2 and HTLV WB profiles: the highest possible number was included due to their low prevalence in previous studies.
- Indeterminate WB profiles: included around 4%–10% of samples for each population type.

Beyond the serological profile, two critical practical criteria were enforced to ensure sample quality and consistency:

1. Sufficient plasma volume: samples needed at least 20 μL of plasma.
2. Minimal freeze-thaw cycling: samples must have been subjected to only one previous freeze-thaw cycle.

**Fig 1:**
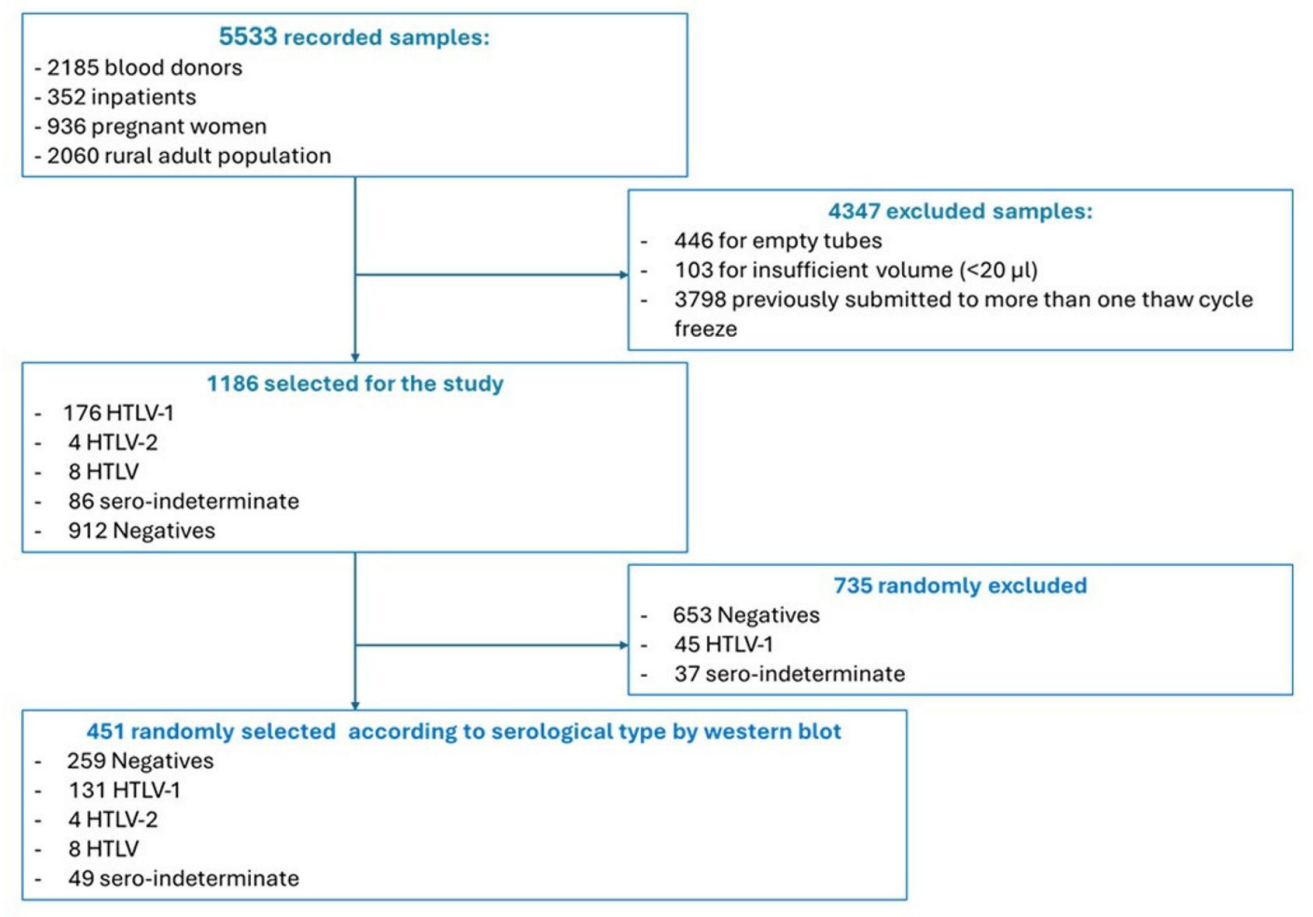
Flowchart about illustrating sample selection and processing.

The flow chart illustrates the sample selection and handling (Fig. 1).

Finally, among the 451 samples included, 131 (29%) were previously confirmed HTLV-1 positive by using HTLV BLOT 2.4, MP diagnostic (WB), while 259/451 (57.4%) were seronegative. The remaining samples (61/451, 13.5%) displayed the following profiles: HTLV-2 (4/61); HTLV (8/61); and indeterminate (49/61). The inpatients included two individuals with ATL, three with TSP/HAM, two with both TSP/HAM and HAIM, and a last one with only HAIM (34) (Table 7).

**TABLE 7:** Types of selected samples according to serological results by western blot (WB)

Samples origin	Total, N (%)	HTLV-1 (%)	HTLV-2 (%)	HTLV (%)	IND (%)	NEG^*a*^ (%)
Blood donors	119 (26.4)	29 (24.4)	1 (0.8)	0 (0)	17 (14.3)	72 (60.5)
Pregnant women	76 (16.9)	20 (26.3)	0 (0)	4 (5.3)	8 (10.5)	44 (57.9)
Inpatients	50 (11.1)	21 (42.0)	0 (0)	2 (4.0)	2 (4.0)	25 (50.0)
Rural adult population	206 (45.7)	61 (29.6)	3 (1.5)	2 (1.0)	22 (10.7)	118 (57.3)
Total	451 (100)	131 (29.0)	4 (0.9)	8 (1.8)	49 (10.9)	259 (57.4)

^*a*^ NEG, corresponds to samples which were negative after using HTLV-I/II ELISA 4.0, MP diagnostic, and WB as well; N, total number of included samples per item; IND, indeterminate profile.

### HTLV-1/2 serological and molecular tests applied to confirm the infection

As previously mentioned, all samples were previously subjected to ELISA test (HTLV-I/II ELISA 4.0, MP diagnostic) for the screening of HTLV-1/2 antibodies in plasma, according to the manufacturer’s instructions. Then, positive samples were further tested by WB assay (HTLV BLOT 2.4, MP Diagnostic) for confirmation and differentiation of HTLV-1 and/or HTLV-2 infection. Furthermore, molecular tests by polymerase chain reactions (PCRs, using DNA extracted from the buffy coat of all positive samples by ELISA) and sequencing performed on fragments of 522 and 210 bp from the Env and Tax/Rex regions, respectively, were used to confirm the infection as well and characterize the HTLV-1 circulating genotypes. For more details on these serological and molecular tests employed, please refer to our previous work (12, 34). In this work, we considered a seronegative at screening ELISA as a true negative as the gold standard. However, samples displayed reactive test results at ELISA with HTLV-1 and HTLV-2 infections confirmed by WB only or both WB and PCR have been considered as true positives. Furthermore, samples with reactivity by ELISA with a negative WB profile or a WB indeterminate profile with a negative PCR were considered samples containing potential cross-reactivity for ASSURE HTLV-I/II Rapid Test and were included to assess the analytical specificity.

### Test principle of ASSURE HTLV-I/II Rapid Test

ASSURE HTLV-I/II Rapid Test is a lateral flow immunochromatographic test using as point of instrument, physical and chemical properties of the colloidal gold nanoparticles (AuNPs) to get simple and rapid detection of total HTLV-1/2 antibodies from serum, plasma, finger-pricked whole blood, or from whole blood with anticoagulant (17). In other words, compared to other metal nanoparticles, AuNPs are known to display a prominent “surface plasmon resonance” property (35–37). Simply, an electromagnetic field from a light source applied to AuNPs in phase leads to an oscillation of conduction electrons on the surface of AuNPs, plasmon waves. The particularity with AuNPs is that these reflected plasmon waves directly correspond to the human visible wavelengths of light (35, 37). In the case of the ASSURE HTLV-I/II Rapid Test, colloidal gold nanoparticles are labeled to a recombinant trifusion HTLV-1/2 antigens (MGK), including GD21 and Rgp46-I, also known as MTA-1 for HTLV-1, and Rgp46-II or K55 glycoprotein for HTLV-2. Briefly, while biotinylated labeled to bovine serum albumin (BSA) will be stripped on the nitrocellulose membrane for the control line, the non-conjugate MGK fragment antigens will be immobilized on the test line (17). When a positive HTLV-1/2 sample is added to the sample well, the chase buffer carries HTLV-1/2 total antibodies that flow upward at the same time that MGK trifusion fragment antigens labeled with colloidal gold and goat anti-biotin are also conjugated with colloidal gold particles. The conjugate goat anti-biotin will recognize the biotinylated-BSA to ensure the integrity of the immunochromatographic assay. While HTLV-1/2 total antibodies will be taken into a sandwich between the MGK fragment antigens on the test line and those being conjugated with colloidal gold. Thus, the accumulation of colloidal gold in the test line due to the presence of MGK fragments, antigens-HTLV-1/2 antibodies complexes, will give a pink-purplish color, allowing us to consider the sample as positive.

### HTLV-1/2 serological analysis with the IC assay

For each sample, we added 10 µL of plasma to the sample well before the addition of 80 µL of chase buffer. The results were read between 15 and 20 min as recommended by the manufacturer. The tests were independently read by two different members of the laboratory (E.F.O. and H.F.M.-M.) before the attribution of “+, ++, +++, and –” when HTLV-1, HTLV-1/2, or HTLV-2 and HTLV samples displayed, respectively, low, medium, strong, or negative reactivity with the IC assay. In the rare case of discordant results, a third member of the laboratory (A.D.) interfered to settle between the two results.

### Statistical analysis

All data were prior organized by using Excel software. Then, specificity and sensitivity, as well as positive and negative predictive values, were calculated as previously described (38, 39). In other words, the PPV was calculated by considering samples with a WB profile HTLV-1 and HTLV-2 as true positives and samples with a positive reactivity by IC assay but a negative result with ELISA, WB, and PCR as false positives (Table 8). Similarly, NPV was calculated by considering our gold standard (seronegative samples by ELISA) as true negatives and samples with WB profile HTLV-1 or HTLV-2 and a negative result with the IC assay as false negatives. Kappa value was also calculated to assess the overall inter-rater agreement about ASSURE HTLV-I/II Rapid Test outcomes, by considering the IC overall results and the gold standard. Furthermore, we calculated a 95% confidence interval (95% CI) to have a variation around the respective obtained proportions. Furthermore, to assess if the shelf life of our samples affected our findings of the IC assay, we also performed a logistic regression test using the RStudio software (2023.09.0+463). However, subgroup analysis is presented by population group and HTLV-1 genotype.

**TABLE 8:** Items selected to calculate the PPV and NPV

	N
True positive (HTLV-1 or HTLV-2)	135
True negative (ELISA −)	218
True positive^*a*^	90
True negative^*b*^	216
False positive^*c*^	2
False negative^*d*^	41

^*a*^ True positive, number of true positive (HTLV-1 or HTLV-2) which displayed a positive reactivity with the IC assay.

^*b*^ True negative, number of samples with a seronegative result by ELISA and the IC assay.

^*c*^ False positive, samples with a positive reactivity by IC assay but a negative result with ELISA, WB, and PCR.

^*d*^ False negative, samples with WB profile HTLV-1 or HTLV-2 and negative result with the IC assay.

## Data Availability

The authors confirm that all relevant data are within the paper. For researchers who meet the criteria for access to confidential data, the data are available on request from the corresponding author.
